# Dance with me? Analyzing interpersonal synchrony and quality of interaction during joint dance

**DOI:** 10.3758/s13428-024-02563-5

**Published:** 2024-12-11

**Authors:** Noemí Grinspun, Eden Landesman, Yonnhatan García, Tal-Chen Rabinowitch

**Affiliations:** 1 The School of Creative Arts Therapies, University of Haifa, Abba Khoushy Ave 199, 3498838 Haifa, Israel; 2 Metropolitan University of Educational Sciences, 7760197 Santiago, Chile; 3 Centro Internacional Iberoamericano, Gordon College of Education, Haifa, Israel

**Keywords:** Interpersonal synchrony, Music, Movement, OpenPose, Wavelet transform, Ecological settings, Mother–child dyad

## Abstract

This methodological paper examines the assessment of interpersonal synchrony during a joint dancing task between mothers and their children (aged 4 to 5 years) using OpenPose. This pose estimation tool captures movement in naturalistic settings. The study analyzes 45 mother–child dyads, comparing two analytical methods for assessing synchrony, and examines their correlation with the Coding Interactive Behavior (CIB) measure of interaction quality. The first method employs cross-wavelet transform (CWT) coherence to assess synchrony based on vertical head movement. This straightforward and computationally efficient approach reveals a significant correlation between interpersonal synchrony and CIB scores, thus implying its potential as a reliable indicator of interaction quality and suggesting its potential as a measure of interaction quality. The second method, the generalized cross-wavelet transform (GCWT), analyzes synchrony across multiple body parts, offering a more complex and detailed analysis of interpersonal dynamics. However, it did not significantly correlate with the CIB scores. Our findings suggest that focusing on head movement using CWT can effectively capture critical elements of interpersonal synchrony linked to interaction quality. In contrast, despite its richness, the more complex GCWT approach may not align as closely with observed interactive behaviors as the CIB scores indicate. This study underscores the need to balance methodological complexity and ecological validity in research, offering insights into selecting analytical techniques based on research objectives and the nuances of interpersonal dynamics. Our results contribute to the field of interpersonal synchrony research, emphasizing the benefits of efficient methods in understanding mother–child interactions and interaction relationships in general.

Dance is a human behavior prevalent in all cultures, appearing often in social contexts and in conjunction with music (Hartmann et al., 2023). The musical experience in dance provides a rich environment for synchronous and coordinated behavior, as when we dance, we tend to synchronize our bodies to the same beat (Patel & Iversen, 2014), and the musical cues encourage the body to move in time with it rhythmically, a phenomenon known as entrainment (Patel & Iversen, 2014). In a social context, dancing with others requires entrainment with the music and with the other person’s movements (Washburn et al., 2014; Cross & Morley, 2008).

## The roles of musical interactions and interpersonal synchrony in children’s development

Our first musical experiences usually occur with our caregivers (Wallace & Harwood, 2018). During infancy, generally, the first exposure to music occurs through lullabies sung by the caregivers (L’Etoile & Shannon, 2006; Trehub et al., 1997). These interactions between infants and caregivers are characterized by their multimodality. This multimodality is manifested when caregivers talk and sing to infants, which often happens in conjunction with touch and movement, for example, when caregivers sing to infants while rocking or bouncing them to the regular beat of the music (Cirelli et al., 2018). Vlismas et al. (2012) proposed that mothers preferred songs and rhymes that also have rhythmic movements when they engage with their infants.

Early parent–child musical interactions have been shown to enhance the mother’s perception of bonding and emotional communication between her and her infant (Creighton, 2011; Wallace & Harwood, 2018). Affiliative bonds are based on behavioral, genetic, hormonal, neural, and mental processes that align to establish the parent–infant bond (Feldman, 2012; Feldman, 2017; Leclère et al., 2014). Bonding is defined as the emotional connection between parents and their children (Joas & Möhler, 2021). Feldman (2007) proposed that parenting is co-constructed between the parent and the child during social interactions and defined it as reciprocal, co-regulated, attuned, mutually influencing, or synchronous. During musical interactions, it is believed that a mother's co-created rhythmical movements and touch with her infant are crucial to the infant's sense of pleasure (Longhi, 2008). These are also essential for a healthy mother–infant relationship and are intrinsic to their bond (Trevarthen & Malloch, 2000).

A significant component of musical interactions is interpersonal synchrony, which is achieved when two or more individuals perform coordinated rhythmic actions with each other (Rabinowitch & Meltzoff, 2017a, 2017b). When people move together in time, they become coordinated with one another in ways that may promote liking and affiliation (Hove & Risen, 2009; Cross et al., 2016; Reddish et al., 2013). From a developmental perspective, interpersonal synchrony is thought to play a key role in acquiring social skills and in experiencing social interactions during the early years of life (Feldman, 2007). Cirelli et al. (2014) showed that 14-month-olds who listened to a song while being bounced by an adult and facing an experimenter who executed knee bends in synchrony with the bouncing baby were more likely to offer help to that experimenter when compared to bouncing in asynchrony with the experimenter’s knee bends. In addition, synchronous interaction can improve social attitudes between interacting children. Rabinowitch and Knafo-Noam (2015) demonstrated that children who participated in a synchronous rhythmic interaction showed enhanced judgments of perceived similarity and closeness to their interacting partner. Tunçgenç and Cohen (2016) showed that synchronous movement produced a significant positive change in bonding between in-group and out-group children. Moreover, interpersonal synchrony was shown to enhance peer cooperation in preschoolers (Rabinowitch & Meltzoff, 2017a).

The preschool age is considered to be a critical stage for children’s social development (Harrist & Waugh, 2002; Nguyen et al., 2021). Indeed, during the preschool age, social interactions expand to individuals outside of the family (Harrist & Waugh, 2002; Nguyen et al., 2021). Harmonious, mutually regulated, and reciprocal synchrony in the parent–preschooler dyad predicts better preschooler integration with their peers (Vizziello et al., 2000; Harrist & Waugh, 2002). Furthermore, during the preschool age, movement synchrony with the mother was found to be related to the child’s expression of pleasure, cooperation, reciprocity in interactions, sense of control, self-efficacy, social involvement, and empathy (Shuper-Engelhard et al., 2021). However, research regarding the direct outcomes of synchronization and affiliative bonding between the caregiver and the preschool child is still lacking (Nguyen et al., 2020; Quiñones-Camacho et al., 2022).

To summarize, a large body of literature shows that both musical and synchronous interactions have short- and long-term effects on children’s social and emotional development. Indeed, capturing and analyzing those interactions in a robust yet efficient and noninvasive way is crucial for the emergence of more studies and analyses. We shall next discuss the ways by which it is possible to capture and measure this highly intricate and complex interaction in the lab, as well as in more ecological settings, such as the home, and provide a sketch of previous studies examining the comparison between movement patterns—and specifically synchronous interactions—and relationship quality.

## Existing methods connecting movement patterns and relationship quality

The relationship between movement and the quality of the interaction was explored by Egmose et al. (2017), who showed a connection between automatically extracted motion features and interaction quality in mother–infant interactions at 4 and 13 months. In that study, the Coding Interactive Behavior (CIB; Feldman, 1998) scale was used to rate the mother–infant interaction quality. Using a motion capture system, the kinetic energy of the upper body, arms, and head motion was calculated and used as segmentation to extract coarse- and fine-grained motion features. Egmose et al. (2017) examined whether some movement patterns, e.g., high or low levels of the mother’s motion, were related to adaptive or aversive interactions between the mother and her infant (Egmose et al., 2017). The interactions were conducted in an observation room inside a lab. However, previous studies have found that mothers and infants interact differently in the laboratory and at home (Jaffe et al., 2001; Egmose et al., 2017). For that reason, Egmose et al. (2017) highlighted the importance of future studies to investigate the relationship between motion features and interaction qualities in more natural and ecological settings, such as the home.

Over the last decade, motion-tracking systems have advanced, offering a more accurate and less time-consuming process for segmenting, annotating, and analyzing movement during an interaction (Romero et al., 2017). However, a far more ecological and natural way to investigate joint dance would be to have the participants dance without any additional tracking devices on their bodies or clothes. The principal advantage of video-based techniques, in comparison with tracking devices, is their simplicity. Unlike a motion capture system, no sensors or markers are required to analyze body movements. Instead, video taken with a simple single camera is sufficient for obtaining the required time series of bodily movements in order to accurately analyze the interaction (Cao et al., 2018; Paxton & Dale, 2013; Ramseyer & Tschacher, 2011). The low-cost and completely wireless motion capture system can, therefore, provide researchers with new tools for exploring social motor coordination as well as for developing innovative treatment strategies (Chang et al., 2011; Chung et al., 2014; Romero et al., 2017).

## Measuring interpersonal synchrony

A substantial portion of the research on interpersonal synchrony has used discrete tasks such as finger tapping (Repp, 2006; Repp & Su, 2013). The finger-tapping task has the advantage of exploring dynamic interactions using straightforward analyses. Nevertheless, this approach generally has limited ecological validity (Toiviainen & Hartmann, 2022) as it only utilizes a very specific and small portion of the movement range. For children, finger tapping and direct instructions on movement may limit their ability to use their natural and spontaneous beat-finding process, thereby restricting their natural sensorimotor repertoire (Almeida et al., 2017). Other studies have explored more ecologically oriented approaches to measure interpersonal synchrony. One such approach involved a storytelling session between a 14-month-old infant and an adult using a motion capture system in a lab setting (Cuadros et al., 2019). Another approach was bouncing (knee bends) while having sensors on the wrists of the experimenters and was carried out in a lab setting (Cirelli et al., 2014). A different approach involved swinging, performed in a lab setting without body sensors but with a laser beam that monitored the movement of the swings themselves (Rabinowitch & Meltzoff, 2017a, 2017b). Finally, the drumming approach was employed in a natural setting, specifically in a daycare center, without body sensors, but with microphones embedded inside the drums themselves (Kirschner & Tomasello, 2009).

When dancing to music, a move-as-you-wish paradigm allows children to make their own free and individual choices of movements when responding to music. Instead of predetermined actions such as finger tapping, bouncing up and down, swinging, or drumming, the sensorimotor repertoire is spontaneously selected based on each child’s specific body capabilities and free in-the-moment choice (Almeida et al., 2017). Dance is a highly ecological movement interaction (Carlson et al., 2018; Toiviainen & Hartmann, 2022), especially when explored outside the lab. Also, the observation of parent–child dyads engaged jointly in free, improvisational dance and movement can show us specific characteristics in the dynamic relations of the dyad (Gavron, 2013; Proulx, 2003; Shuper-Engelhard et al., 2021), which are often nonverbal and unconscious and can provide rich information about the quality of the relationship (Gavron & Mayseless, 2018; Shuper-Engelhard et al., 2021).

## Wavelet approach for interpersonal synchrony research

Different methods have been used to analyze interpersonal synchrony based on the time–frequency domain (Fujiwara & Yokomitsu, 2021; Fujiwara & Daibo, 2016). The cross-wavelet transform (CWT) is a powerful analytical technique that examines the time–frequency relationship between two time series. Unlike traditional methods that may only capture linear relationships, CWT allows researchers to identify localized correlations in both the time and frequency domains, providing a more detailed view of the synchrony between signals. By transforming the data into a joint time–frequency space, CWT can reveal the presence of shared oscillatory behavior, highlighting both amplitude and phase interactions over time. This dual analysis can uncover intricate patterns of synchrony that might be missed by traditional linear methods (Issartel et al., 2015; Fujiwara & Daibo, 2016; Fujiwara & Yokomitsu, 2021). The CWT method generates a cross-wavelet power spectrum, which illustrates the regions of high common power, and a phase difference spectrum, which depicts the relative phase relationship between the signals (Issartel et al., 2015). These outputs enable the identification of specific frequency bands and time intervals where the two signals exhibit significant synchrony, offering insights into the underlying mechanisms driving their interaction. This makes CWT particularly useful for studying complex systems where interactions are dynamic and nonstationary, such as physiological processes, brain activity, and social interactions.

On the other hand, the generalized cross-wavelet transform (GCWT) involves calculating bivariate cross-wavelet transforms between all possible pairs of individual signal components in X and Y, resulting in a total of N × M cross-wavelet transforms. The GCWT method involves performing a continuous wavelet transform on each set of movement data dimensions. This is followed by evaluating the global amplitude and phase relationship of the two multidimensional time series for each time–frequency point. This is achieved by estimating the distribution of the pairwise continuous wavelet transforms in the complex plane (Chavez & Cazelles, 2019; Soon et al., 2014). Then, for each point in the time–frequency plane, the distribution of the N × M cross-transform values is modeled using a bivariate normal distribution in the complex plane (Toiviainen & Hartmann, 2022). The size and shape of the estimated distribution, specifically the variance and eccentricity, offer a comprehensive measure of the degree of time- and frequency-localized synchrony between the multivariate time series. A distribution with a large major axis indicates strong synchrony. Additionally, the angle between the distribution’s major axis and the real axis of the complex plane provides a comprehensive measure of the mutual phase difference between the multivariate time series (Toiviainen & Hartmann, 2022). The GCWT is similar to the bivariate CWT but GCWT does not differentiate between in-phase and anti-phase movement. This aligns with multivariate time-domain methods like canonical correlation analysis and partial least squares correlation, which do not consider the sign of correlation in their latent space projections (Toiviainen & Hartmann, 2022).

## Using head versus whole-body key points in wavelet analyses

A major difference between the two types of analysis is the use of the data from the head only in the vertical axis (i.e., CWT) versus data from the whole body (i.e., GCWT). It has been proposed that the GCWT is helpful for different kinds of analysis, such as electroencephalography (EEG; Chávez et al., 2019) and general movement data (Toiviainen & Hartmann, 2022). For example, Toiviainen and Hartmann (2022) used the GCWT for coordinated movement analysis, where they applied the analysis to motion capture data derived from a mirror game and dance movement tasks.

As for the CWT analysis, it seems that although there is only one key point being measured, it may be sufficient, as vertical head movements tend to follow the rhythm of the music spontaneously (Burger et al., 2013), and gross body movements, such as vertical movements, have been implicated as a key source of co-performer interaction in previous work (Williamon & Davidson, 2002; Eerola, et al., 2018). Hence, in a recent study, Bigand et al. (2024) found that during interpersonal synchrony, bounce movements may be more relevant for capturing the rhythmic interaction than movements in other directions (Bigand et al., 2024). They observed that head bobs or the head movement on the anteroposterior axis tend to synchronize through music. In contrast, full-body lateral movements synchronize through visual contact with the other participant, but not with the music. Interestingly, only vertical movements or bouncing allow synchronization with the music and with the other participant from the dyad, suggesting a distinct role in facilitating interpersonal synchrony. Additionally, Bigand et al. (2024) emphasize that bouncing triggers multiple sensory feedback signals, which can strengthen internal timekeeping and, consequently, interpersonal coordination (Quirko, 2024; Bigand et al., 2024). Thus, for the purpose of the current study, we propose that analyzing the cross-wavelet transform (CWT) of the data on the vertical movement of the heads from a dyad during dancing could be a more effective and simple method for predicting the quality of their interaction in contrast to a more complex analysis of movement, such as the GCWT.

To this end, we videotaped an interaction during a free dancing-together task between a mother and her preschool child without using body sensors or other technological equipment. We then analyzed the level of interpersonal synchrony between mothers and children during the dancing task using computer vision, which is now feasible due to the rapid development of artificial intelligence technology (Zhu, 2021), in two different ways: The first involved an analysis of movement in 25 body key points, and the second involved an analysis of the head key point only. Each of these coding schemes would lead to a different analysis method path: The coding of multiple key points would serve as a base for the GCWT analysis, whereas the coding of the head key point by itself would lead to the CWT analysis. To support our argument, we illustrate the correlations between both analyses of the interpersonal synchrony between the mother and her child during the dancing-together interaction (i.e., CWT and GCWT) with a well-established mother–child interaction measure, the CIB scale (Feldman, 1998). No previous study to date has explored (a) the differences in the level of interpersonal synchrony when analyzing the head versus whole body using such techniques as wavelet transform, and (b) compared both head and whole-body methodological analyses to the interactants’ interaction quality to see which one correlates better with the level of interpersonal synchrony.

## Materials and methods

### Participants

To determine the sample size for our study, we conducted an a priori power analysis using G*Power (Faul et al., 2007). Our goal was to perform a two-tailed test with an alpha value of 0.05 and a beta value of 0.1. This was based on a Pearson correlation coefficient of 0.5 that we obtained from a pilot study, which indicated a relationship between our operationalization of interpersonal synchrony (i.e., “coherence”) and the CIB “Elaboration” subscale (Feldman, 1998; see below). Our analysis showed that a sample size of 37 participants would give us a power of 0.9. Therefore, we recruited 45 dyads for our study to avoid any data loss issues and possible attrition. The study included 45 mothers (mean age = 38.3 years, *SD* = 3.9) and their typically developing preschool-age children (17 boys and 28 girls; mean age = 4.8 years, *SD* = 0.4). Participants were recruited through social media advertisements as well as the through the University of Haifa’s recruitment system.

### Procedure

#### Dancing-together task

We evaluated the dyads (comprising the mother and her preschool child) in their homes, where they were asked to dance together. Each video had a duration of 3 minutes, captured at a frame rate of 30 frames per second. In the dancing-together task, we used a 3-minute instrumental piece specifically composed for this research project, featuring a tempo of 120 beats per minute. This tempo was selected to align with the spontaneous motor tempo (SMT) typically exhibited by children aged 4 to 5 years (Eerola et al., 2006). The task guidelines instructed the participants to “move freely together with the music for 3 minutes.”

#### Pose extraction

**Fig. 1:**
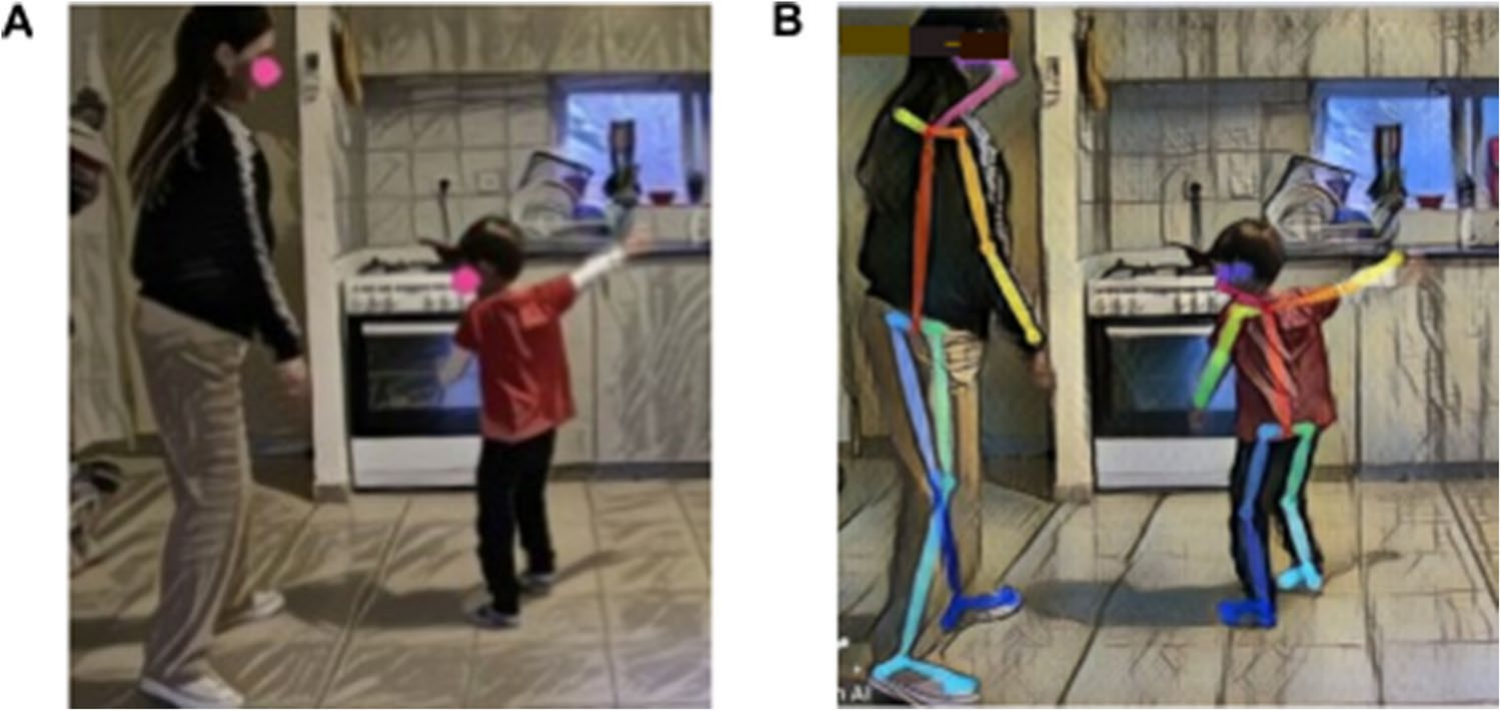
**A** An illustrated example of the head key point extracted with OpenPose during the dancing-together task recorded by the researchers at the participants’ home. **B** An illustrated example of the 25 key points extracted with OpenPose during the dancing-together task recorded by the researchers at the participants’ home

We extracted skeletal information from the videos using the deep learning-based multi-person two dimensional (2D) pose estimator OpenPose (Cao et al., 2018). It uses a simple 2D camera input not requiring any external markers or sensors. It also allows the analysis of videos to be performed, including variations in resolution and settings (Fujiwara & Yokomitsu, 2021; Kojovic et al., 2021). We performed both CWT and GCWT analyses. For the CWT analysis, we selected the head key point from the OpenPose output (Fig. 1A). For the GCWT, we selected key points from the head, mid-hip, and hands from 25 points altogether (Fig. 1B).

Since the output of OpenPose is in JSON format, we used an R code to convert the output into one .csv file (de Jonge-Hoekstra & Repgen, 2020). This .csv file was then imported to MATLAB (The Math Works, Inc., 2022). For missing data, we interpolated it using MATLAB’s interp1 (The Math Works, Inc., 2022). All codes used in this paper are also available in our repository: https://github.com/ynerpoe/Dancing_Methods.

### Interpersonal synchrony analysis using the cross-wavelet transform (CWT) method

We performed the data analysis using two types of analyses, both centered on the time–frequency domain. For the CWT analysis, we calculated the head position in the *x* and *y* axes (in centimeters) over time (in seconds). Since the head bounce occurs in the sagittal plane or on the *y*-axis, we plotted the position of the head in centimeters along the *y*-axis versus time.

Firstly, we performed a cross-wavelet analysis method involving each time series’ spectral decomposition. This allows for the examination of the degree and pattern of synchronization at each component signal frequency and analyzes two time series together more effectively, thus providing a more accurate outcome (Grinsted et al., 2004; Issartel et al., 2006; Khan & Yener, 2018). More precisely, it evaluates the cross-spectrum of two time series across time and can show how the time-localized coherence and relative phase differ at different frequency ranges (Fujiwara et al., 2021; Schmidt et al., 2014). Coherence measurement through cross-spectrum analysis examines the proportion of variance at each frequency component in a series of one individual that can then be predicted by the other individual’s series, forming an index of the degree of interpersonal synchrony between interactants at each frequency component (Fujiwara & Daibo, 2016; Schmidt et al., 2012; Schmidt & O’Brien, 1997). This type of analysis has the advantage of not requiring constant properties (i.e., stationarity) in each time series. It is better than short-time-windowed techniques because of its multiscale properties. The wavelet approach allows for precisely detecting properties in a very complex signal, and it can be used for a wide range of motor signals (Fujiwara & Yokomitsu, 2021; Fujiwara et al. 2023; Issartel et al., 2006, 2015), such as in the case of free dance, where it allows the decomposition of a signal into its frequency components while preserving temporal information (Sun et al., 2023). The CWT analysis measures the similarity between two time series at each frequency component, indicating the degree of interpersonal synchrony, which is usually shown through colors ranging from blue (no synchrony) to warmer colors (perfect synchrony; Richardson et al., 2007; Schmidt & O’Brien, 1997; Fujiwara et al., 2020a, 2020b). In the context of interpersonal synchrony, a value of 0.21 indicates a low level of synchrony between two individuals, suggesting that their movements are not closely aligned. Conversely, a higher value of 0.44, for example, reflects greater synchronization, implying that their actions are more closely coordinated. These values help quantify the degree of synchrony between individuals, with higher numbers indicating more robust interpersonal synchrony. For this study, we employed MATLAB (MathWorks, 2022) and the wavelet toolbox (Grinsted et al., 2004) to calculate the cross-wavelet coherence for each dyad’s time series.

**Fig. 2:**
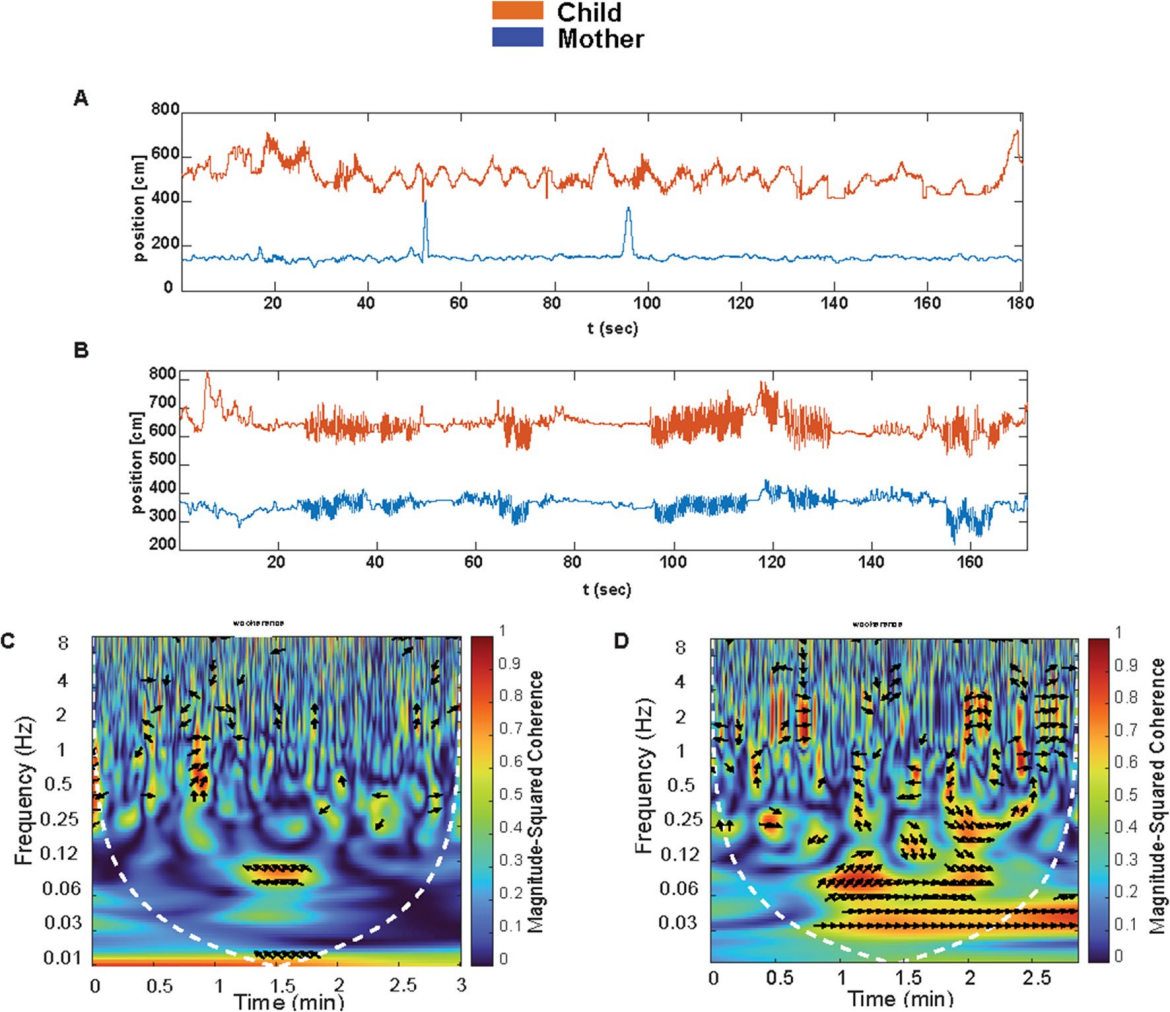
Two examples of low and high coherence between the mother and the child during the dancing-together task analyzed with the CWT method. *Note*. **A** An example of low coherence of head movements of the mother and the child on the *y*-axis during the dancing-together task. **B** An example of high coherence of head movements of the mother and the child on the *y*-axis during the dancing-together task. **C** An example of low wavelet coherence between the head movements of the mother and the child. The *y*-axis represents each normalized frequency/frame, the *x*-axis represents the time in frames. The magnitude of the cross-wavelet coherence (WTC) is represented by color. Mean coherence = .22. **D** An example of high wavelet coherence between the head movements of the mother and the child on the *y*-axis. The *y*-axis represents each normalized frequency/frame, the *x*-axis represents the time in frames. Mean coherence = .33

Figure 2 shows an example of the CWT coherence analysis from two different videos, where the first one (Fig. 2A, C) portrays less interpersonal synchrony between the child and the mother (coherence = .22) than the second video (Fig. 2B, D; coherence = .33). For the cross-wavelet coherence plots (Fig. 2C, D), warmer colors (red) represent regions with significant interrelation, while colder colors (blue) signify lower dependency between the series. The arrows in the wavelet coherence plots (Fig. 2C, D) represent the series’ lead–lag phase relations. A zero-phase difference means the two time series move together on a particular scale. Arrows point to the right or left when the time series are in phase or anti-phase. When the two series are in phase, it indicates that they move in the same direction, and anti-phase means that they move in the opposite direction. Arrows pointing to the right-down or left-up indicate that the first variable (child) is leading, while arrows pointing to the right-up or left-down show that the second variable (mother) is leading. In both videos, both the mother and the child were leading at different moments when they were synchronized, as shown by the arrow’s direction.

### Interpersonal synchrony analysis using the generalized cross-wavelet transform (GCWT) method

To perform another type of analysis also based on the frequency domain but including different body points from the mother and the child estimated with OpenPose, we applied a generalized cross-wavelet transform (GCWT) method. Subsequently, we computed the magnitude of the generalized cross-spectrum and real/imaginary parts of the results. For the context of interpersonal synchrony during free dancing, a vector magnitude of 871.012 for example, indicates lower synchrony; conversely, a magnitude of 3250.620 for example, indicates greater synchrony.

**Fig. 3:**
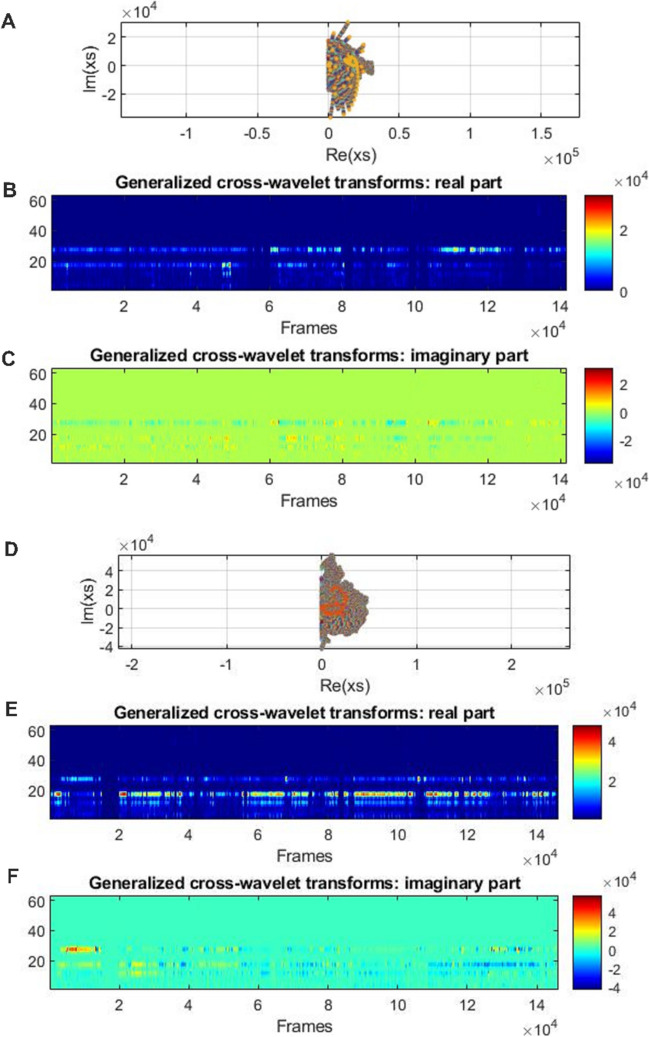
Two examples of low and high magnitude of generalized cross spectrum from the mother and the child during the dancing-together task analyzed with GCWT. *Note*. **A** An example of low magnitude of generalized cross spectrum from head movements of the mother and the child on the *x*- and *y*-axis during the dancing-together task. **B** Real part of generalized cross-wavelet transforms from data of the mother and the child on the *x*- and *y*-axis during the dancing-together task from the dyad with low synchrony. **C** Imaginary part of generalized cross-wavelet transforms from data of the mother and the child on the *x*- and *y*-axis during the dancing-together task from the dyad with low synchrony. **D** An example of high magnitude of generalized cross spectrum of the mother and the child on the *x*- and *y*-axis during the dancing-together task. **E** Real part of generalized cross-wavelet transforms from data of the mother and the child on the *x*- and *y*-axis during the dancing-together task from the dyad with low synchrony. **F** Imaginary part of generalized cross-wavelet transforms from data of the mother and the child on the *x*- and *y*-axis during the dancing-together task from the dyad with low synchrony

Figure 3 shows an example of the analysis from two videos, where the first one shows a lower magnitude of the vector which indicates weaker synchrony (Fig. 3A; mean magnitude from the vector = 871.012), while a large major axis indicates strong synchrony **(**Fig. 3D; mean magnitude from the vector = 1289.90; Toiviainen & Hartmann, 2022). We performed the analysis with a MATLAB routine (https://github.com/ynerpoe/Dancing_Methods) using the MATLAB functions for the calculation of wavelet tensor (Toiviainen & Hartmann, 2022) and for the calculation of generalized cross-wavelet transform (Toiviainen & Hartmann, 2022).

#### Global rating for the quality of the mother–child interaction

To evaluate the quality of the interaction, we performed an analysis using the Coding Interactive Behavior (CIB; Feldman, 1998) over the 3 minutes of the dancing-together task. CIB includes 21 codes for parents, 16 codes for children, six dyadic behavioral codes, and lead–lag relationship codes rated on a scale from 1 (minimum) to 5 (maximum). The scales used in this study were selected for the nature of the task to assess both the overall dynamics and progression of the session, and the interactive engagement and individual styles of each participant.

Altogether, we selected seven codes that were the most relevant to the relationship dynamics that we were interested in comparing in this study (two for the parent, two for the child, and three for the dyad; see description below). A trained researcher who is well-experienced in CIB coding completed the scoring. A second trained and well-experienced researcher scored 20% of the videos. Interrater reliability (IRR) with the second scorer was calculated using the intraclass correlation coefficient (ICC). The IRR was high (89–100% agreement per video).

Here, we describe the definition of the quality of each of the seven interaction scales we used in our study (CIB; Feldman, 1998).

Mother codes:

- *Acknowledging*: This scale measures the mother’s attentiveness to her child’s social signals as can be seen through gaze, facial expressions, or body movements. A score of 1 indicates no attentiveness and a score of 5 indicates consistent attentiveness.
- *Elaborating*: This scale measures whether the parent expands and elaborates the child’s actions or verbal communication. A score of 1 indicates the absence or very limited occurrence of such elaboration. A score of 5 indicates that the parent consistently elaborates the child’s actions or signals.

Child codes:

- *Child initiation:* This scale measures whether the child initiates mutual activities and expects the parent to follow his/her lead. A rating of 1 indicates the minimal degree of child initiation, whereas a rating of 5 indicates that the child frequently initiates activities and communicative efforts.
- *Joint attention*: This scale measures whether the child’s gaze is consistently focused on the parent or on their joint activity. A rating of 1 indicates that the child’s gaze is averted and is not focused on the parent or on a joint object of attention. A rating of 5 indicates that the child looks at the parent or at a joint object throughout the interaction.

Dyadic codes:

- *Dyadic adaptation regulation*: This scale measures how well parents and children adjust their level of interaction based on the signals of their partner. A rating of 1 indicates that the interaction is not mutually regulated and adaptive. A rating of 5 indicates that the interaction is mutually regulated and adaptive.
- *Dyadic fluency*: This scale measures the rhythm and flow of the interaction. A rating of 1 indicates that the interaction is not fluent. The lack of flow may be due to either an anxious and highly labile exchange or a withdrawn and apathetic interaction. A rating of 5 indicates that the interaction is fluent and smooth.
- *Dyadic reciprocity*: This scale measures whether the mother and the child are involved in a “give-and-take” interaction or not. A score of 1 indicates that no reciprocity has been observed. A rating of 5 indicates that the dyad engages in reciprocal interaction, smoothly moving and responding to each other’s cues and frequently engaging in give-and-take play.

## Results

### Interpersonal synchrony analysis validation

**Fig. 4:**
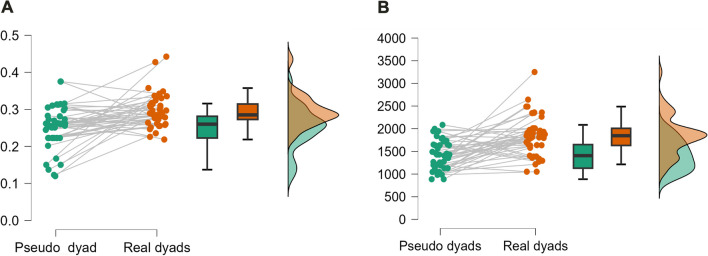
**A** Coherence for pseudo-dyads and real dyads for the CWT analysis. Coherence for real dyads (marked in red rainclouds, box and violin plot) is significantly greater than the coherence for pseudo-dyads (marked in green raincloud, box and violin plot), *p* < .001. **B** Magnitude of the vector for pseudo-dyads and real dyads for the GCWT analysis. Magnitude of the vector for real dyads (marked in red rainclouds, box and violin plot) is significantly greater than the magnitude of the vector for pseudo-dyads (marked in green raincloud, box and violin plot)

To assess the level of synchrony in a real-time series, we created randomly shuffled virtual data from all the 45 dyads participating in this study. Then, we calculated both the CWT and GCWT of the real dyads and the pseudo-dyads. Subsequently, we employed a two-tailed paired-samples *t*-test for 45 real dyads versus 45 pseudo-dyads. For the CWT, results showed that the mean coherence for real dyads (*M* = .30, *SD* = .04) was significantly higher than the mean coherence for the pseudo-dyads (*M* = .25, *SD* = .05; *p* < .001, *t* = −7.801), suggesting that participants spontaneously synchronized movement with each other during the dancing-together task significantly more so than the pseudo-dyads’ data (Fig. 4A). For the GCWT, we also found a difference between the pseudo-dyads (*M* = 1408.449, *SD* = 333.317) and the real dyads (*M* = 1841.940, *SD* = 437.983; *p* < .001, *t* = −5.223) (Fig. 4B**)**, demonstrating that interpersonal synchrony for the 45 dyads is not merely a result of chance.

### Cross-wavelet transform (CWT) analysis for interpersonal synchrony

We performed the cross-wavelet analysis for the head position at the *y*-axis measured with OpenPose. The results were obtained from analyzing the 3-minute duration of the dancing-together task for the 45 dyads. As measures of interpersonal synchrony (see above), we calculated the coherence values for each of the mother–child dyads. For all 45 dyads, we obtained coherence values that ranged between 0.21 and 0.44, with a mean value of 0.29 and a standard deviation of 0.05.

### Generalized cross-wavelet transform (GCWT) analysis for interpersonal synchrony

We conducted a GCWT analysis on the positional data of 25 points along both the *x*-axis and *y*-axis, as measured using OpenPose. This analysis was performed over a 3-minute period during a dancing-together task, involving 45 dyads. For each dyad, we calculated the mean magnitudes of the vectors derived from all 25 points. The magnitudes across the 45 dyads ranged from 871.012 to 3250.620, with an average value of 1847.196 and a standard deviation of 451.349. These values provide insight into the varying degrees of synchrony among the dyads during the task. A large major axis indicates strong synchrony, while a small axis indicates weaker synchrony.

### Interpersonal synchrony and the quality of the interaction

**Fig. 5:**
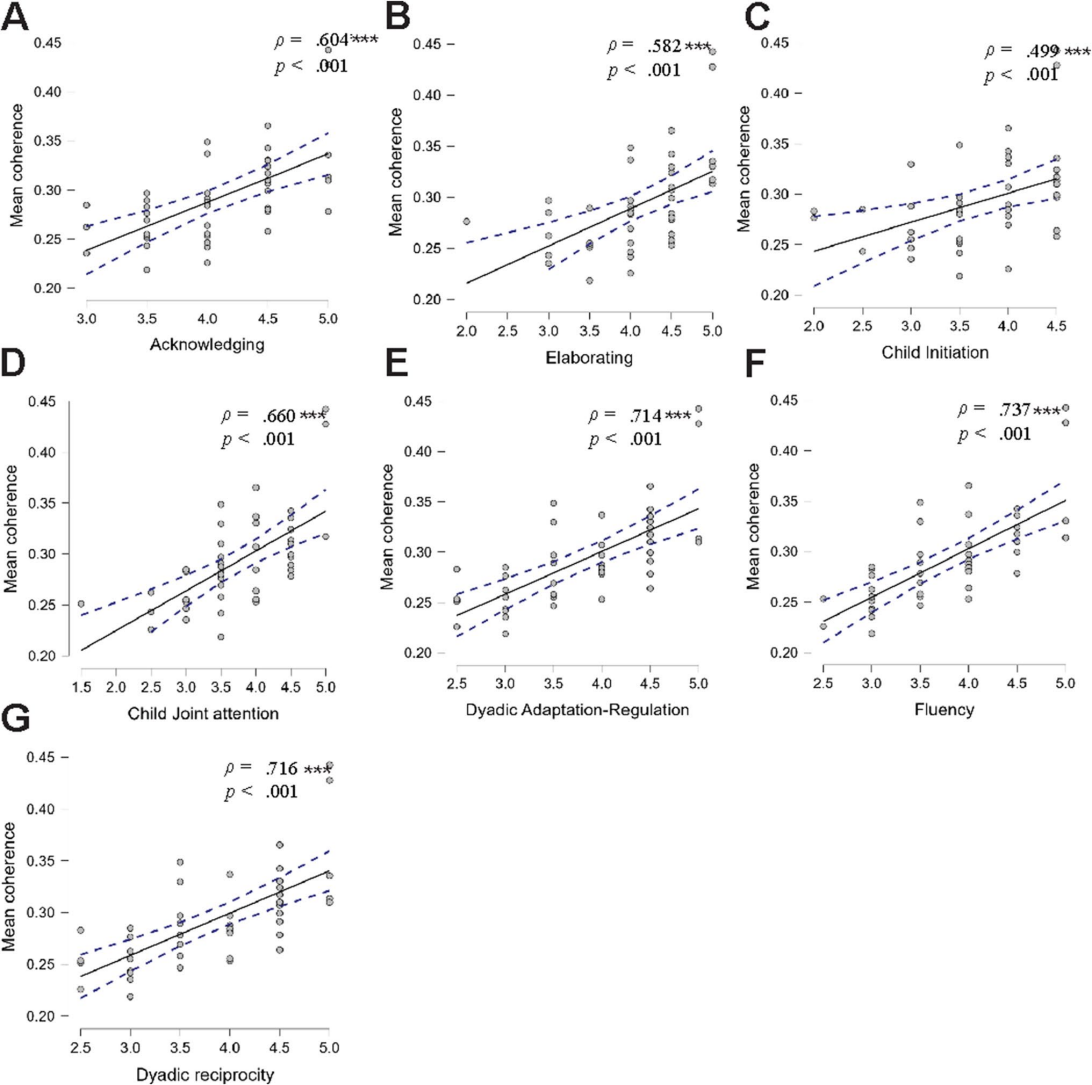
Scatter plots of the correlations between coherence scores and each of the CIB scales. *Note.* Spearman’s correlation coefficient and statistical significance are noted in each panel. *** *p* < .001

For the CWT analysis, dyads with a higher coherence score during the dancing-together task also had better quality of interaction measured by the Coding Interactive Behavior (CIB) scales. A Spearman correlation analysis examined the relationship between the coherence values and the quality of interaction scale scores. A significant positive correlation was found between coherence and *acknowledging* (*ρ* = .604, *p* < .001; (Fig. 5A), coherence and *elaborating* (*ρ* = .582, *p* < .001; (Fig. 5B), coherence and *child initiation* (*ρ* = .499, *p* < .001; (Fig. 5C), coherence and *child joint attention* (*ρ* = .660, *p* < .001; (Fig. 5D), coherence and *dyadic adaptation regulation* (*ρ* = .714, *p* < .001; (Fig. 5E), coherence and *dyadic reciprocity* (*ρ* = .737, *p* < .001; (Fig. 5F), and coherence and *fluency* (*ρ* = .716, *p* < .001; Fig. 5G).

For the GCWT, after calculating the average magnitude of the vector and correlating it with the quality of the interaction, no significant correlations were found with *acknowledging* (*ρ* = −.152, *p* = .318), *elaborating* (*ρ* = −.110, *p* = .472), *child initiation* (*ρ* = −.019, *p* = .901), *child joint attention* (*ρ* = −.054, *p* = .722), *dyadic adaptation regulation* (*ρ* = −.096, *p* = .528), *dyadic reciprocity* (*ρ* = −.054, *p* = .523), or *fluency* (*ρ* = −.100, *p* = .514).

Additionally, a Spearman correlation analysis between the two methods for measuring interpersonal synchrony (CWT and GCWT) did not yield significant results (*ρ* = −.035, *p* = .818).

## Discussion

We compared two different analyses: the first involved an analysis of movement in different body key points, and the second involved an analysis of the head vertical movement only. These analyses were meant to examine the synchronization between two individuals, a mother and her preschool child, during a free dance task in the home environment. We selected a free dancing-together task because it represents a natural way to interact cross-culturally (Tarr et al., 2014) and for its significant role in fostering social bonding (Dunbar, 2012; Tarr et al., 2014). When humans hear music, they naturally synchronize their movements to the beat, which can lead to the experience of positive emotions (Phillips-Silver et al., 2010; Trost et al., 2017; Basso et al., 2021). Therefore, dance offers a unique platform for experiencing and learning about interpersonal synchrony and specific characteristics of the relational dynamics between the dancing individuals (Gavron, 2013; Proulx, 2003; Shuper-Engelhard et al., 2021). Observing and analyzing nonverbal elements during a dance can provide valuable insights into the dynamics of a dyadic relationship (Gavron & Mayseless, 2018; Shuper-Engelhard et al., 2021). Dancing involves a complex interplay of various interpersonal coordination factors, including touch, eye contact, sensory–motor interactions, synchronized movements, physical coordination, and emotional expressions (Basso et al., 2021; Sofianidis et al., 2012; Washburn et al., 2014). Moreover, nonverbal elements during dancing can convey information through bodily gestures, movement, proximity, posture, and appearance (Hanna, 2012).

It has been demonstrated that dancing together can offer insights into the interaction between parents and their children. It can also provide information on various emotional and behavioral disorders, self-regulation (Shuper-Engelhard et al., 2021), and developmental issues. Hence, we consider it crucial to further investigate the study of interpersonal synchrony through movement analysis to contribute to and improve existing methods. This study examined the effectiveness of two analytical methods, the cross-wavelet transform (CWT) and the generalized cross-wavelet transform (GCWT), in evaluating interpersonal synchrony during mother–child dancing tasks. Our findings emphasize the importance of selecting the right approach to accurately capture key elements of interaction quality, as evidenced by the Coding Interactive Behavior (CIB) scores. Our results showed that the CWT analysis, which was calculated based on the vertical head movement or “bounce,” was significantly correlated with the quality of the mother–child interaction as reflected in the CIB scores, as opposed to the GCWT analysis, which was calculated based on multiple body parts, which yielded no significant correlations between the level of interpersonal synchrony and mother–child quality of interaction. These findings suggest that CWT effectively captures essential synchrony aspects that indicate interaction quality. Thus, a single, straightforward metric—head bounce—seems to be a powerful and efficient proxy for evaluating interpersonal dynamics, aligning with prior research emphasizing the role of rhythmic movement in synchrony and social bonding. It has been demonstrated that different types of movements synchronize in different ways: for example, anteroposterior movements such as head bobs synchronize through music, while hand gestures and full-body lateral movements synchronize through visual contact (Bigand et al., 2024). Only one specific movement during dance, the vertical movement, represents a supramodal pacesetter of coordination, synchronizing through music and visual contact and at the pace of the musical beat (Bigand et al., 2024). In their 2024 study, Bigand et al. demonstrated that vertical bounce has unique characteristics. Interestingly, simply listening to the same music or only seeing the partner had a similar synchronizing effect as when both factors (listening to the same music and seeing each other) were present. Second, they observed that the magnitude of the bounce increased when participants could see each other. Lastly, they noted that bounce was the only movement to synchronize with a specific temporal periodicity, matching the musical beat. These findings suggest that bounce may have a distinct role in facilitating interpersonal synchrony, and that bounce is a fundamental component of locomotion (Troje, 2002; Bigand et al., 2024), which is one of the first isochronous signals infants experience through maternal walking (Larsson et al., 2019).

Indeed, during the dancing-together task, we found a strong and positive correlation between the head bounce coherence and the mother’s CIB scales, *acknowledging* and *elaborating*. This suggests that mothers who are more in tune with their child’s cues are also more synchronized with them during the movement task, adjusting their movements accordingly. These findings align with the idea that interpersonal synchrony between a mother and her child is a critical aspect of attentive caregiving (Abraham et al., 2014; Leclère et al., 2014). In mother–infant interaction, when the mother coordinates her movements with her infant, she demonstrates awareness and a desire to participate in the infant’s experience (Stern, 2004; Egmose et al., 2017).

As for the child CIB scales, we found that the head bounce coherence and *child joint attention* had a positive and significant correlation, as well as head bounce coherence and *child initiation*. This means that there is a correlation between the mother–child synchrony and the level of the child’s attention to the mother during the joint activity, initiating mutual activities with the expectation that the mother follows his/or her lead. Indeed, Fitzpatrick et al. (2017) conducted a study that found a positive correlation between performance during spontaneous synchrony in a hand-clapping game with an experimenter and joint attention.

In addition, we found a positive correlation between head bounce coherence and *dyadic adaptation regulation*, *dyadic reciprocity*, and *fluency*, which are all dyadic CIB scales. These findings provide additional strong support to the credibility of our head bounce, or CWT coherence data analysis, as the more the mother and the child work together as a single, harmonious unit: adapting, regulating, and experiencing reciprocity and fluency with one another, the higher the coherence score is.

In contrast, the GCWT method, which utilized movement data from multiple body parts, did not correlate significantly with any of the CIB scales. This lack of correlation may be attributed to the complexity and richness of the data, which, while providing a comprehensive picture of movement, may not directly map onto the specific elements of interaction quality assessed by CIB. This highlights a critical consideration for researchers: while multidimensional analyses can offer detailed insights into movement patterns, they may obscure the core synchrony components most relevant to understanding interaction quality.

The discrepancy between these two analyses emphasizes the need to balance methodological complexity with ecological validity. While GCWT offers a detailed analysis of interpersonal dynamics, its failure to align with observed interactive behaviors suggests that complexity alone does not guarantee a better measurement of synchrony. Instead, the simplicity and directness of the CWT analysis, focusing on one key movement characteristic, appear more effective in reflecting the quality of mother–child interactions.

This study contributes to the broader field of interpersonal synchrony research by advocating for the judicious selection of analytical techniques based on research objectives. More straightforward methods like CWT may be preferable when the goal is to capture core elements of interaction quality, as is often the case in developmental and behavioral studies. This approach enhances the ecological validity of the findings and ensures that the analysis remains accessible and interpretable.

Future research should continue to explore the application of different analytical methods to various interaction contexts, considering both the benefits and limitations of each approach. By prioritizing methods that balance complexity with ecological validity, researchers can better understand the nuances of interpersonal dynamics, particularly in naturalistic settings. Additionally, integrating qualitative assessments with quantitative metrics could further enrich our understanding of how synchrony influences and reflects the quality of social interactions.

Our analysis method accurately and efficiently assesses interpersonal synchrony while minimizing intrusiveness and enhancing the ecological validity of the research setting. The results of the study could significantly contribute to the interdisciplinary field of interaction analysis. In conclusion, this study underscores the value of efficient, targeted methods in synchrony research, highlighting the significance of head movement as a key indicator of interaction quality. These insights have implications for both theoretical and practical models of interpersonal synchrony, offering a pathway to more effectively assess and understand the dynamics of mother–child interactions.

## Data Availability

Data are available on https://github.com/ynerpoe/Dancing_Methods. The study was not preregistered.

## Code availability

The analysis scripts for this paper are publicly available on https://github.com/ynerpoe/Dancing_Methods.
